# Exploring the Role of Peroxisome Proliferator-Activated Receptors and Endothelial Dysfunction in Metabolic Dysfunction-Associated Steatotic Liver Disease

**DOI:** 10.3390/cells13242055

**Published:** 2024-12-12

**Authors:** Ana Paula Madariaga Traconis, Misael Uribe-Esquivel, Varenka Julieta Barbero Becerra

**Affiliations:** 1 Translational Research Unit, Medica Sur Clinic & Foundation, Mexico City 14050, Mexico; a.madariagatraconis@my.ula.edu.mx; 2 Latin American University, Cuernavaca Campus, Mexico City 62290, Mexico; 3 Digestive Diseases Unit, Medica Sur Clinic & Foundation, Mexico City 14050, Mexico; muribe@medicasur.org.mx

**Keywords:** PPAR, MASLD, endothelial dysfunction, liver, inflammation, pharmacological targeting

## Abstract

The endothelium is a well known regulator of vascular homeostasis. Several factors can influence the balance of the bioavailability of active substances. This imbalance can lead to inflammation and, consequently, endothelial dysfunction, which is an underlying pathology in cardiovascular disease that commonly coexists with metabolic and chronic diseases such as metabolic dysfunction-associated steatotic liver disease (MASLD). In MASLD, a reduction in nitric oxide availability is observed, and as a result, hepatic stellate cells and liver sinusoidal endothelial cells are activated. Considering the extensive research dedicated to finding several targets with diagnostic and therapeutic effects, nuclear hormone receptors such as peroxisome proliferator-activated receptors have been highlighted as being highly influential in the gut–liver–adipose axis and are considered potential regulators of metabolism and inflammation in several pathologies. Currently, PPAR agonists are widely explored in clinical trials and experimental studies. Agents such as lanifibranor, elafibranor, daidzein, and Icariin have shown promise in improving the metabolic, hepatic, and cardiovascular health of patients with MASLD. This review aims to provide a comprehensive overview of the role of peroxisome proliferator-activated receptors in endothelial dysfunction and MASLD, exploring their mechanisms in disease progression and potential pharmacological targeting.

## 1. Introduction

The endothelium plays a crucial role in vascular hemostasis, acting as a barrier between the blood and the vessel wall. It also functions as a signaling hub, modulating endothelial function and preventing platelet adhesion and blood clot formation by producing various substances that influence platelet behavior [1]. Disruptions in the metabolic balance of endothelial mediators can lead to endothelial dysfunction (ED), which predisposes the vasculature to inflammation, increased permeability, and the development of arteriosclerosis, platelet aggregation, and thrombosis [2]. Conditions such as hyperglycemia and insulin resistance (IR) can further compromise endothelial function by initiating molecular interactions that impair the endothelial response [3]. A specialized form of endothelium, known as liver sinusoidal endothelial cells (LSECs), accounts for about 20% of the total hepatic cell population. These cells have a distinct anatomical structure and play a key role in maintaining liver function [4]. In response to liver injury, LSECs regenerate and modulate fibrosis by secreting angiocrine factors [5]. However, in chronic liver diseases, such as metabolic-associated fatty liver disease (MASLD), LSECs undergo “capillarization”, a process that leads to the formation of a continuous endothelial layer [6].

This is a hallmark of chronic liver diseases, and is commonly associated with conditions like dyslipidemia, obesity, type 2 diabetes mellitus (T2DM), and IR [3]. ED in MASLD is often driven by IR, which disrupts several pathways, including the stimulation of nitric oxide (NO) production by the endothelium [7]. NO plays a critical role in vasodilation, and its reduced availability can lead to impaired blood flow. Conversely, pre-existing ED may contribute to MASLD by activating hepatic stellate cells (HSCs), a process exacerbated by reduced NO availability [8].

Peroxisome proliferator-activated receptors (PPARs) are a family of nuclear hormone receptors that include three isoforms: PPARα, PPARβ/δ, and PPARγ [9]. PPARs are essential regulators of inflammation, lipid metabolism, and fibrogenesis [10]. Given their pleiotropic effects, PPARs are critical in regulating not only glucose and fatty acid metabolism but also in modulating inflammatory responses and fibrosis [11]. Furthermore, due to their involvement in lipid and glucose metabolism, as well as in the regulation of inflammation and fibrosis, PPARs are increasingly being recognized as important therapeutic targets for treating metabolic diseases. In this review, we will explore the potential of PPARs as pharmacological targets in the pathogenesis of MASLD.

## 2. The Function of the Endothelium

The endothelium is a monolayer of cobblestone-shaped cells that cover the inner wall of blood vessels. It is a crucial regulator of vascular homeostasis that acts as a barrier between the blood and blood vessels and serves as a signaling activator that modifies the phenotype of the endothelial wall through changes in permeability, inflammation, vascular tone, and injury repair [12]. The endothelium is <0.2 µm thick and weighs approximately 1 kg in an average-sized human, covering a total surface area of 4000 to 7000 m^2^ [13].

### 2.1. Endothelial Glycocalyx

The endothelium contains a glycocalyx, which is a complex gel between flowing blood and the endothelial wall [14]. Its composition and dimensions fluctuate as it continuously replaces material sheared by flowing plasma [15]. Primarily, the endothelial glycocalyx (EG) is composed of proteoglycans (PGs), glycosaminoglycans (GAGs), and glycoproteins (GPs). These result in a negative charge, which acts as a physical barrier that prevents direct contact between cells and molecules on the endothelial surface [16]. PGs are key components of the EG, consisting of a core protein attached to GAG chains. The two main types of PGs in the EG are syndecans and glypicans [17].

Syndecans are transmembrane proteins present on the surface of most cells in the body. There are four known syndecans in vertebrates, but the EG primarily contains syndecan-1 (SDC1), featuring extracellular, transmembrane, and cytosolic domains [18], which allow them to bind GAGs and respond to external signals that are transduced into the intracellular environment, such as shear stress [19]. On the other hand, glypicans are not transmembrane proteins; nevertheless, they are attached to the luminal membrane of endothelial cells by a glycosylphosphatidylinositol anchor [20]. There are six known glypicans in mammals, with glyplican-1 being the only one expressed in the endothelium that specifically binds to GAGs [21]. Its ectodomain specifically binds to GAG heparan sulfate, and its anchor molecule is believed to be positioned near lipid rafts and caveolae. Caveolae are membrane structures abundant in signaling molecules that act as communication centers in the cell membrane. This positioning enables glypicans to engage in various signaling pathways with cytokines and other substances, including the vasodilator nitric oxide (NO) [19].

For their part, GAGs are linear polysaccharides that do not branch, consisting of 20 to 200 repeating disaccharide units. They represent the most abundant component of the extracellular matrix (ECM) [18]. The five primary types of GAGs are heparan sulfate (HS), chondroitin sulfate (CS), dermatan sulfate (DS), keratan sulfate, and hyaluronan (HA). While all five GAGs are found in the ECM, they are not evenly distributed. HS is the most abundant, accounting for 50–90% and appearing in a 4:1 ratio with CS, the second most common GAG [22].

The biosynthesis of the core proteins syndecan and glypican occurs on ribosomes attached to the membrane. After synthesis, the core protein is moved into the lumen of the endoplasmic reticulum and then to the Golgi apparatus, where GAG side chains are attached, polymerized, and sulfated. The core protein, along with the GAGs, is subsequently transported to the cell surface, where it is either integrated into the cell membrane, as seen with syndecans, or linked to the surface via an anchor molecule, such as in the case of glypican. Unlike other GAGs, HA is synthesized directly on the cell membrane and does not attach to a core protein [23].

Finally, GP is situated on the surface of endothelial cells and is covered by the extracellular glycocalyx in healthy conditions. In contrast to PGs, GPs do not interact with long-chain GAGs; instead, they feature short, branched oligosaccharide units that are covalently bonded [24]. Endothelial GPs function as membrane-bound cell adhesion molecules and are classified into three families based on their structural and functional attributes: selectins, immunoglobulins, and integrins [25]. Integrins facilitate the interaction between platelets and endothelial cells (ECs) by binding to collagen and laminin in the subendothelial matrix [26]. Immunoglobulins, such as intercellular adhesion molecule-1 and -2 (ICAM-1,-2), vascular cell adhesion molecule-1 (VCAM-1), and platelet/endothelial cell adhesion molecule-1, primarily mediate inflammatory cell adhesion to the endothelium by binding to the target cell’s integrins [27].

These GPs are vital for the proper recruitment of leukocytes, which involves a sequence of regulated steps: rolling, adhesion, and transmigration. This process allows neutrophils, monocytes, eosinophils, and some lymphocytes to move through the endothelium by binding to their respective integrins [28].

The key selectins in the extracellular glycocalyx are P-selectin and E-selectin. Both are important for the initial adhesion of leukocytes and platelets to activated ECs [25]. E-selectin is exclusively found on the endothelium and binds to interleukin-1 (IL-1), tumor necrosis factor-α, or lipopolysaccharides; meanwhile, P-selectin is present on both ECs and platelets and binds to histamine or thrombin. However, unlike P-selectin, which is stored, E-selectin is inducible and requires transcription, translation, and movement to the cell surface for its function [29].

The functions of these proteins, in addition to providing a negative charge to repel molecules, also include regulating vascular permeability, hemostasis, blood viscosity, and the inflammatory response [30]. The glycocalyx itself is physiologically inert; however, the addition of both plasma- and endothelial-derived soluble factors makes it physiologically active. The interaction between ECs and coagulation factors, plasma molecules, and inflammatory cells through various adhesion molecules within the glycocalyx is essential for the proper functioning of hemostasis, blood viscosity, and inflammatory responses [24].

### 2.2. Endothelial Functions

In healthy blood vessels, the interaction between ECs and blood will not induce platelet adherence and clot formation, as this is an active process. Platelets circulate in a quiescent state throughout the vascular system until required. As a result, ECs create a protective anticoagulant and antithrombogenic layer, actively producing substances that modulate platelet behavior [1]. One key substance is NO, which ECs continuously synthesize from L-arginine using the endothelial isoform of nitric oxide synthase (eNOS) in its membrane-bound form [31]. NO can easily diffuse across cell membranes and enter nearby platelets, activating guanylate cyclase (GC). This activation transforms guanosine triphosphate (GTP) into cyclic guanosine monophosphate (cGMP). The increase in cGMP results in vasodilatation, which interferes with the release of stored intracellular calcium (Ca^2+^). This suppression reduces platelet activation and aggregation [32], inhibiting the proliferation and migration of vascular smooth cells, preventing leukocyte adhesion, and limiting oxidative phosphorylation in the mitochondria [16].

Various metabolic changes may disrupt the delicate balance of endothelial mediators, leading to endothelial dysfunction (ED); this condition can be defined as an imbalance in the bioavailability of active substances originating from the endothelium. This leads to a predisposition to inflammation and increased vascular permeability, and can facilitate the development of arteriosclerosis, platelet aggregation, and thrombosis [2]. Conditions such as hyperglycemia and insulin resistance initiate molecular interactions that compromise endothelial function. These interactions lead to increased vascular tone, enhanced vascular permeability, oxidative stress, and inflammatory responses, resulting in greater arterial stiffness, impaired vascular remodeling, endothelial activation, and, ultimately, the development of atherosclerosis [3].

Key physiological features for ED include the reduced availability of endothelial NO, decreased endothelium-mediated vasodilation, dysregulation of hemodynamics, impaired fibrinolytic activity, increased expression of adhesion molecules and inflammatory genes, heightened oxidative stress, and greater endothelial permeability [33].

## 3. Metabolic Dysfunction-Associated Steatotic Liver Disease and Endothelial Dysfunction

### 3.1. MASLD Epidemiology and Pathogenesis

Some years ago, non-alcoholic fatty liver disease (NAFLD) became the leading cause of chronic liver disease worldwide; it now currently affects 38% of the adult population [34]. This disorder is expected to become the leading cause of liver transplantation worldwide by 2030 [35]. The prevalence of NAFLD is expected to rise over the next decade, paralleling the global epidemics of obesity and type 2 diabetes mellitus [36]. Significant progress has been made in the past 10 years in understanding the complex pathophysiological mechanisms underlying this widespread liver condition. NAFLD has been recognized as a multisystem disease where insulin resistance and associated metabolic dysfunction contribute to its development and serious liver-related complications, including cirrhosis, liver failure, hepatocellular carcinoma, and extrahepatic issues such as cardiovascular disease [37].

Later, in 2020, international experts proposed a change in terminology for NAFLD to metabolic dysfunction-associated fatty liver disease (MAFLD) [38]. However, in 2023, three multinational liver associations proposed renaming it again, this time from MAFLD to metabolic dysfunction-associated steatotic liver disease (MASLD) [39] to emphasize the disease as an independent entity without exclusion criteria, highlighting that MASLD can coexist with other chronic liver diseases [38]. MASLD is defined by metabolic dysfunction as its foundation, underlining its significant impact on disease and its reduced heterogeneity. Key criteria include being overweight or obese and having type 2 diabetes mellitus (T2DM) [40]. In this context, chronic inflammation plays a central role and is marked by the accumulation of bioactive lipids, lipotoxicity, oxidative stress, and the secretion of proinflammatory molecules [41,42]. This diagnosis is evaluated via either imaging or liver biopsy in patients presenting one of the following five cardiovascular risk factors: arterial hypertension, hypertriglyceridemia, low plasma HDL cholesterol, increased body mass index (BMI) or waist circumference, and increased fasting serum glucose levels. Other causes of chronic liver disease like viral hepatitis or excessive alcohol consumption (>30 or 20 g per day for men and women, respectively) are absent [43]. The most common exogenic trigger of MASLD is overnutrition, expanding adipose tissues, and driving ectopic fat deposits, which cause changes in tissue metabolism and dysregulation, for example, insulin resistance in hepatocytes [44].

The accumulation of fat is considered an early step in the origin of the disease [45]. With dietary constituents acting as pivotal drivers of the disorder, the overconsumption of nutrients crucially involves weight gain, disruption of the gut microbiome, and metabolic dysregulation as early steps and risk factors of MASLD [46]. The increased consumption of sugars, especially fructose, which is added as a sweetener to beverages and processed foods [47], induces lipogenesis in hepatocytes and fatty acid synthesis. This occurs by providing essential substrates and regulating the expression of key enzymes involved in lipid metabolism via the transcription factors sterol response element-binding protein 1c (SREBP1) and carbohydrate-responsive element-binding protein (ChREBP) [48]. The accumulation of lipids, predominantly triglycerides, impairs fatty acid oxidation. Altered lipid export from the liver will initiate a series of harmful effects known as lipotoxicity, which fuels inflammatory processes and the progression of MASLD [49]. Hepatic free cholesterol interacts with a transcriptional regulator that induces cell proliferation and reprogramming called yes-associated protein (YAP) and the transcriptional coactivator with PDZ-binding motif (TAZ), also known as YAP-TAZ. Through interaction with YAP-TAZ, free cholesterol will facilitate the lipotoxic effects of this transcriptional regulator, promoting tissue inflammation [50].

### 3.2. The Participation of Liver Sinusoidal Endothelial Cells in MASLD

As previously mentioned, the vascular endothelium participates in multiple physiological and pathophysiological mechanisms, such as vascular tone, inflammation, and platelet function, among others [51]. Nonetheless, there is a specialized and phenotypically differentiated endothelium with a distinctive anatomical location and structure called liver sinusoidal endothelial cells (LSECs) [52]. LSECs represent 20% of the total number of hepatic cells from the non-parenchymal group of cells placed in an interface between the hepatic parenchyma and the blood from the hepatic artery and portal vein [4]. In response to liver injury, LSECs respond by regenerating and balancing fibrosis through the secretion of angiocrine factors [5]. Through this abnormal activation, LSECs not only alter their own physicochemical properties but also disrupt their communication with hepatic stellate cells (HSCs) and hepatocytes, which collectively aggravates the process of fibrosis and can promote the progression from MASLD to MASH [53,54]. In chronic liver diseases, such as MASLD, LSECs undergo a process known as capillarization, where the fenestrae disappear, leading to the formation of a continuous endothelial layer, which is a common indicator of chronic liver disease and is hypothesized to be the first stage in liver fibrosis [6]. In addition, it can directly contribute to increased hepatic vascular resistance through the enhanced activation of the cyclooxygenase-1-thromboxane vasoconstrictor pathway [55].

### 3.3. The Relationship Between Endothelial Dysfunction and MASLD

Capillarization commonly coexists with cardiovascular risk factors, such as dyslipidemia, obesity, and T2DM, all of which are related to the presence of insulin resistance (IR) [3]. Thus, the underlying pathology in cardiovascular disease is ED [56]; this process also involves several key cellular mechanisms. Initially, under typical conditions, HSCs remain quiescent [57]. However, chronic inflammation can activate these cells, leading to the excessive production of collagen type 1, which contributes to liver damage [14,58,59]. As HSCs become activated and contribute to liver damage, LSECs act as a barrier that separates hepatocytes and the space of Disse from the sinusoidal lumen. Moreover, they play an important role in delivering nutrients to hepatocytes and removing waste products from the sinusoidal lumen and bloodstream [60].

Clinical evidence has proposed that there is a synergistic effect between fatty liver and overweight conditions in the development of ischemic heart disease [56,61]. This is characterized by endothelial injury, which leads to dysfunction and serves as the initiating event in atherosclerosis. This dysfunction plays an important role in the ischemic manifestations associated with coronary disease [62]. ED in MASLD can result from IR, which disrupts multiple pathways involving the stimulation of NO production from the endothelium, leading to vasodilation and increased blood. This ultimately causes damage to the vascular endothelium and atherosclerosis. Additionally, the secretion of endothelin-1, which serves as a vasoconstrictor, is a key factor contributing to both IR and ED [7]. Indeed, ED is commonly observed in patients with conventional cardiovascular risk factors, including diabetes mellitus, dyslipidemia, obesity, and smoking; it is significantly correlated with the development and progression of atherosclerosis [63]. On the other hand, long-term hyperglycemia induces alterations and the degradation of heparan sulfate from the glycocalyx. In addition, elevated levels of reactive oxygen species associated with hyperglycemia not only degrade glycosaminoglycans on the glycocalyx, but also activate matrix metalloproteinases, leading to the proteolysis of these sugar chains; this was studied recently by Zhang and colleges in order to evaluate the progression of diabetic nephropathy. They observed that this process results in glycocalyx shedding and promotes kidney disease in diabetic patients. Furthermore, hyperglycemia enhances the endocytic recruitment of fatty acid-binding protein 1, a coactivator of the PPAR pathway, thereby regulating the activation of intracellular PPAR signaling [64]. A similar situation may be occurring in a liver scenario due to the presence of MASLD; however, this area of study has not been explored yet.

According to clinical studies, a meta-analysis involving 34,043 patients reported that patients with MASLD have a 64% higher risk of developing major cardiovascular events compared to patients without MASLD [65]. Because the protective effects of the endothelium may be lost, this leads to a negative prognosis for patients with cardiovascular disease [66].

An inverse mechanism has also been suggested, where pre-existing ED may contribute to MASLD due to reduced NO availability leading to the activation of HSCs [8]; however, the exact cellular and molecular mechanisms are still unknown. Moreover, although various non-invasive methods have been used to detect hepatic fatty infiltration, liver biopsy is still considered the gold standard for diagnosing MASLD [67]. The search for several targets with potential diagnostic and therapeutic effects has been extensive, as nuclear receptors are considered eminent regulators of energy metabolism and inflammation in several pathologies.

## 4. Nuclear Receptors: Peroxisome Proliferator-Activated Receptors

Peroxisome proliferator-activated receptors (PPARs) are a family of transcription factors that belong to the superfamily of nuclear hormone receptors and consist of three isoforms: PPARα, PPARβ/δ, and PPARγ [9]. PPARs have a modular structure with five functional domains, including an N-terminal region, DNA-binding domain, flexible hinge region, and ligand-binding domain with a large secondary structure containing 13α and a β helices. The ligand-binding domain is the site at which the receptor is activated or inhibited. The fifth domain is the C-terminal [68]. These receptors directly interact and respond to ligands, such as steroids, thyroid hormones, retinoids, cholesterol by-products, lipids, and haem. Additionally, they contain A/B regions that are not well conserved; however, in some instances, these regions function as strong transcriptional activators, engage in direct interactions with other receptor domains, and provide sites of protein phosphorylation. There have been multiple structural studies of nuclear receptors; however, there has been no successful visualization of any intact nuclear receptor [69].

PPARα is located in chromosome 22q12-13.1. It is expressed in tissues with a high rate of acid oxidation—predominantly the liver, skeletal muscle, heart, and brown adipose tissue—with the main function of managing energy metabolism based on nutritional conditions such as fasting and feeding [70,71]. Notably, in the liver, its expression has also been documented in sinusoidal endothelial cells at a lower level in mice and HSCs [72]. In this regard, it has been demonstrated that oleoylethanolamide, an endocannabinoid-like molecule, attenuates the progress of liver fibrosis by blocking HSC activation through PPARα activation [73]. Moreover, hepatic fibrosis caused by arsenic trioxide induces the activation of HSCs through PPARα activation and autophagy, where taurine supplementation alleviates this response [74].

Saturated and unsaturated fatty acids, eicosanoids, and leukotriene B4 are primary endogenous ligands for PPARα. The fatty acid derivative, 1-palmitoyl-2-oleoyl-sn-glycerol-3-phosphocholine, is recognized as an endogenous ligand for PPARα, which plays a protective role against hepatic steatosis [75]. Nevertheless, the main exogenous PPARα agonists are fibrate drugs such as fenofibrate, bezafibrate, and ciprofibrate, which belong to the class of amphipathic carboxylic acids [70].

The role of PPARα in the liver can be understood through both short- and long-term regulatory functions. In the short-term, it initiates early under fasting conditions to facilitate free fatty acid oxidation for cellular energy requirements; meanwhile, in the long-term, it addresses excessive oxidation and the increased production of ketone bodies by regulating lipoprotein and lipogenesis metabolism through the PPARα itself and its ligands. The metabolic pathway depends on the free fatty acid levels in the liver [76].

On the other hand, PPARβ/δ is located on chromosome 6p21.2-21.1, is expressed in skeletal muscle, and has been widely studied both in vivo and in vitro. It acts as a major regulator of glucose metabolism, and it is a promoter of lipid uptake as an energy source for ATP production during fasting and exercise through the mitochondrial β-oxidation pathway [70,77]. Additionally, PPARβ/δ influences plasma lipid levels by regulating fatty acid oxidation and overseeing the handling of glucose in the muscle and liver. Saturated and unsaturated fatty acids, 15-Hydroxyeicosatetraenoic acid, and components of VLDL are endogenous ligands for PPARβ/δ [78]. One of the functions of PPARβ/δ is angiogenesis [79]; it regulates both physiologically and pathological angiogenesis, where PPARβ/δ activates angiopoietin-like 4 (ANGPTL4), a secretory protein that participates in angiogenesis, cancer progression, and metastasis [80]; several other molecules besides ANGPTL4 are activated, such as platelet-derived growth factor receptor beta (Pdgfrb), platelet-derived growth factor subunit B (Pdgfb), and the tyrosinkinase KIT (c-Kit) [81]. When PPARβ/δ is activated by natural ligands, such as prostacyclin I2, or exogenous synthetic ligands, such as GW501516, it induces EC proliferation and angiogenesis, inhibits EC apoptosis, and stimulates the proliferation of human breast and prostate cancer cell lines [82].

PPARγ is located on chromosome 3p25. Specifically, PPARγ1 is expressed in a variety of cells, including immune and brain cells, while PPARγ2 is abundant in brown and white adipose tissue, regulating adipocyte differentiation and lipid metabolism [70,83]. Unsaturated fatty acids, prostaglandin J2, and multiple metabolites serve as endogenous ligands for PPARγ [84]; meanwhile, exogenous ligands are principally thiazolidinediones, which help in reducing IR and hypercholesterolemia; therefore, it promotes a reduction in the late vascular complications of diabetes mellitus [70]. Specifically in the liver, PPARγ promotes the fatty acid-binding protein 4 (FABP4) mediated by free fatty acid (FFA) uptake, which increases the expression of fatty acid synthase and enhances triglyceride accumulation in hepatocytes [85]. Additionally, PPARγ increases the transcription of sterol regulatory element-binding protein-1c (SREBP-1c), which activates other adipogenic genes and converts pyruvate to fatty acids [10].

Among these isoforms, PPARα could be considered the main key regulator of lipid metabolism. It governs a range of processes, including numerous genes involved in fatty acid uptake and activation, mitochondrial and peroxisomal fatty acid oxidation, ketogenesis, lipid droplet biology, and triglyceride turnover. Additionally, it plays a role in glucose metabolism, homeostasis, and managing glycerol for gluconeogenesis [86]. These receptors have pleiotropic actions that make them critical regulators not only in glucose and fatty acid metabolism but also in inflammation and fibrogenesis [11]. Due to these effects, dual-PPARα/δ agonists have been used, demonstrating potent impacts on IR, hyperglycemia, and dyslipidemia in patients with obesity, which will be discussed later.

The clinical efficacy and development of selective PPARβ/δ have been insufficiently researched. Current evidence suggests that the activation of PPARβ/δ may have oncogenic potential, raising concerns about the clinical development and safety of PPARβ/δ agonists. Two Phase II trials were initiated to investigate the effects of GW677954 in patients with diabetes; however, due to the carcinogenicity of the drugs in animal studies, the trials were prematurely terminated, leading to the discontinuation of the development of PPARβ/δ agonists [87].

In terms of PPARγ, it was demonstrated that uncoupling protein 1 (UCP1) transcription is induced through catecholamine-induced cAMP signaling and PPARγ activation in brown/beige adipocytes thus demonstrating its role in lipid metabolism [88]. In general, the use of PPARγ agonists over type 2 diabetes was one of the earliest applications built upon the discovery and knowledge of PPARγ with clinical evidence as an antidiabetic agent, with approved experimental agonists including pioglitazone, rosiglitazone, and rivoglitazone [89].

### 4.1. PPARs and Inflammation

All PPARs are crucial regulators of inflammation, with existing evidence demonstrating that PPARα plays a key role in controlling hepatic inflammation [90]. One of the main anti-inflammatory mechanisms of PPARα is the downregulation of acute-phase genes, including IL-1 receptor agonists (IL-Ra) and nuclear factor kappa B (NF-kB) inhibitors [91]. PPARα primarily regulates inflammation through the transrepression mechanism, binding to NF-kB components and thereby suppressing their transcriptional activity. A study conducted by Pawlak et al. revealed that mice with a mutation in the DNA-binding domain (DBD) of PPARα, which limits its transcriptional activity to transrepression, are protected from liver inflammation and do not develop liver fibrosis in a dietary-induced metabolic-associated steatohepatitis (MASH) model [92]. Additionally, PPARα modulates the duration of inflammation by regulating the catabolism of its ligand leukotriene B4, a chemotactic agent involved in the inflammatory response [93].

In relation to the anti-inflammatory effects of PPARβ/δ, it plays a role in regulating the activation of Kupffer cells (KCs). In the presence of IL-4 and IL-13 stimulation, PPARβ/δ is essential for activating these cells into the macrophage type 2 subtype, which exhibits anti-inflammatory activity. Additionally, it is involved in HSC activation through a signal-transducing factor, leading to HSC proliferation in an acute and chronic liver inflammation event and the expression of CD36, which codes for a membrane receptor that facilitates fatty acid uptake [94]. PPARβ/δ conduces mono-unsaturated fatty acids (MUFAs) production by stearoyl-CoA desaturase 1 (Scd1) upregulation. This process avoids lipotoxicity by increasing fatty acid oxidation, inhibiting fatty acid-induced cytotoxicity in hepatocytes [95]. Furthermore, anti-apoptotic effects were shown in a hepatic ischemia/reperfusion injury model through the inhibition of the NF-κβ pathway in hepatocytes and KCs [96].

On the other hand, PPARγ is considered a potent anti-inflammatory agent. It participates in inflammatory signaling by interacting with inflammatory transcription factors, such as NF-κβ, signal transducer and activator of transcription (STAT), and AP-1; with this activation, it reduces the expression of proinflammatory cytokines [85]. In addition to PPARβ/δ, PPARγ can induce macrophage type 2 polarization [97] and inhibit genes encoding inflammatory molecules while activating the expression of anti-inflammatory mediators to promote anti-inflammatory effects [98].

PPARα and γ are beneficial in medical conditions where inflammation is a major driving force of disease exacerbation, such as MASH [87]. Furthermore, PPARγ’s anti-inflammatory role has been demonstrated in several diseases. For example, it acts as a protector against renal inflammation [99]; in neuronal intoxication with the aid of a neuroprotective flavonoid, diosmin, which has antioxidant and anti-inflammatory effects mediated through PPARγ upregulation [100]; and in chronic inflammation diseases such as atherosclerosis through monocyte inflammation modulation [101].

### 4.2. PPARs and Endothelium

**Figure 1:**
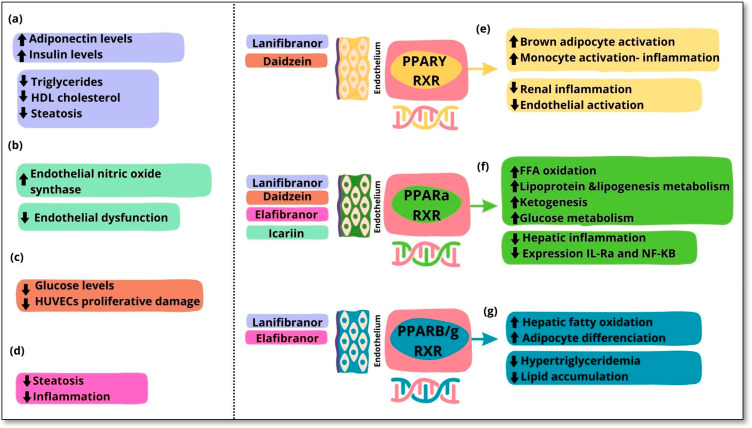
**Biological effects of PPAR isotype agonists related to endothelial dysfunction.** Several PPAR agonists are used as pharmacological targets. The main biological actions of lanifibranor are the improvement in triglycerides, HDL cholesterol levels, and steatosis in addition to the increase in adiponectin and insulin levels (**a**). Icariin, a PPARα agonist, promotes endothelial nitric oxide synthase and improves endothelial dysfunction (**b**). Daidzein, a PPARα and PPARγ agonist, contributes to reducing high glucose levels and ameliorating proliferative damage in human umbilical vein endothelial cells (HUVECs) (**c**). Elafibranor, a PPARα and PPARβ/δ agonist, improves steatosis and inflammation (**d**). The biological effects of PPAR isotypes are as follows: PPARγ is responsible for the activation of brown adipose tissue and monocytes in response to inflammation, contrary to the prevention of renal inflammation and endothelial activation (**e**). PPARα is principally involved in various liver functions, such as the promotion of free fatty acid oxidation, lipogenesis, and glucose metabolism, but it decreases inflammation and the expression of IL-1 receptor agonists (IL-Ra) and nuclear factor kappa B (NF-kB) (**f**). PPARβ/δ promotes the increase in hepatic fatty acid oxidation and adipocyte differentiation while reducing hypertriglyceridemia and lipid accumulation (**g**).

All three PPAR subtypes can promote eNOS activation. For example, fibrates boost nitric oxide synthesis by increasing eNOS expression, stabilizing its mRNA, and activating eNOS through the PI3K, MAPK, and AMPK pathways [102]. PPAR β/δ and PPARγ also play a role in modulating eNOS activity via the PI3K-Akt pathway [103]. The enhancement of eNOS activation and stability via PPARγ is also supported by various intermediates, including heat shock protein 90, adiponectin, and Src homology region 2-containing protein tyrosine phosphatase 2 [104]. Collectively, the influence of PPARs on eNOS and nitric oxide production provides a foundation for using PPAR agonists in clinical settings for cardiovascular disease and hypertension (Figure 1).

LSECs communicate closely with HSCs through NO synthesis to regulate intrahepatic blood and subsequently induce vasodilation. In chronic liver diseases, LSECs lose their specialized phenotype, and their ability to produce NO decreases; meanwhile, HSCs reduce their sensitivity, leading to microvascular dysfunction. All of these events promote intrahepatic vascular liver resistance, which results in portal hypertension development [105]. Furthermore, factors secreted by HSCs influence phenotypic LSEC alterations. These changes in LSECs occur in the early stages of the development of MASLD, often preceding the activation of KCs and HSCs [106].

It has been seen that PPAR agonists may prevent the recruitment and activation of immune cells and confer a vasoprotective phenotype to endothelial cells [105]. It was reported that PPARα and LSECs also help keep HSCs quiescent through extracellular vesicle secretion [58,59]. PPARγ is essential in preventing endothelial dysfunction associated with aging [107], as impaired endothelial PPARγ causes age-related vascular dysfunction [108].

PPARγ activation can contribute to regulating endothelial activation, NO activity, oxidative stress, and apoptosis. To improve endothelial dysfunction, PPARα and PPARγ activation suppresses activator protein-1 (AP-1), which is responsible for increasing the expression of vascular cell adhesion molecule-1 (VCAM-1) in endothelial cells [109]. By inhibiting AP-1, PPARs can decrease the expression of endothelin-1 [110], a powerful vasoconstrictor peptide released by endothelial cells with a proinflammatory effect. This affects cell proliferation and migration due to the activation of PPARα; as a result, endothelin-1 secretion is reduced, decreasing the release of adhesion molecules and reducing monocyte chemotaxis [111].

PPARα receptors can also be activated by a variety of natural and synthetic ligands, including fibrates [112]. The beneficial impact of fenofibrate on vascular function might be partly attributed to enhanced endothelial NO availability because PPARα activation has been shown to increase NO production in endothelial cells [102].

PPARs were identified as playing an important role in lipid and glucose metabolism and being key regulators in metabolic diseases, inflammation, and fibrosis [85]; as a result, they may be a promising pharmacological target in MASLD. In this respect, it is important to mention that the crosstalk between the different PPAR isotypes has been poorly reported. Wahli et al. proposed that the presence of compensatory mechanisms between PPAR isotypes may be an important issue to consider when PPAR agonists are tested. Furthermore, although all three PPAR isotypes are involved in lipid and glucose metabolism, PPARα is considered the master regulator of hepatic lipid catabolism. PPARγ promotes IR, while PPARβ/δ’s role is still unclear; however, it is well known to promote hepatic glucose and fatty acid synthesis [113].

## 5. PPARs as Pharmacological Targets

The significant role of PPAR and other PPAR isoforms underscores the potential of PPARs as pharmacological targets in MASLD (Table 1). Targeting all three PPAR isoforms to address the full spectrum of MASLD, ranging from insulin resistance to liver fibrosis, holds promise [114]. One approach was demonstrated in Phase II clinical trials by Francque et al. using the pan-PPAR agonist lanifibranor. With 95% confidence intervals, lanifibranor has shown potential in improving both metabolic and hepatic health in MASLD via significant improvements in triglycerides, HDL cholesterol, insulin levels, and steatosis, regardless of the diabetes status of the patient [115]. A more recent study by Cooreman et al. focused on improving cardiometabolic health in patients with MASH. In the original analysis, hepatic steatosis was assessed using both imaging and histological analyses for all patients. The improvements in liver histological endpoints included the resolution of MASH, which was defined as a ballooning grade of 0 and lobular inflammation < 1. Additionally, imaging was performed using elastography with a Fibroscan device to ensure that there was no worsening of fibrosis. Furthermore, with the improvement in portal hypertension, most individuals with prediabetes, defined as fasting glucose levels between 5.6 and 6.9 mmol (100 to 125 mg/dL), achieved normal glucose levels (70 to 90 mg/dL). Additionally, there was a significant improvement in cardiometabolic markers, which are associated with an improvement in hepatic and cardiovascular metabolic health, including adiponectin levels, lipid profile, glycemic control, blood pressure, and systemic inflammation. This study also reported 95% confidence intervals. The patients experienced an average weight gain of 2.5 kg; however, it was observed that the increase was related to diet failure and that 51% of patients had stable weight. Furthermore, the therapeutic benefits were noted irrespective of weight changes. These findings indicate that the effect of lanifibranor in MASH is also linked to improved cardiovascular health; nevertheless, gastrointestinal adverse events and peripheral edema and anemia occurred more frequently with lanifibranor than with the placebo [116].

According to a study by Yao et al., the activation of PPARα enhanced vascular endothelial function by decreasing endoplasmic reticulum stress and stimulating endothelial NO synthase in a murine model with streptozotocin-induced diabetes. Their study suggested that a flavonoid glycoside known as *icariin* could achieve these effects by normalizing endoplasmic reticulum stress and regulating the PPARα/Sirt1/AMPKα pathway [117].

One drug studied for its wide range of health benefits is daidzein, a primary isoflavone. Das et al. reported that daidzein had a positive effect on T2DM-related dyslipidemia and vascular inflammation [118]. In 2024, Yang et al. also explored the efficacy of daidzein against high levels of glucose in human umbilical vein endothelial cells, demonstrating that daidzein could ameliorate the proliferative damage in human umbilical vein endothelial cells induced by high glucose levels. This was mediated by the activation of PPARα and PPARγ, suggesting that they might act as dual agonists [119].

Furthermore, a Phase III clinical trial for MASLD treatment is underway, focusing on a dual agonist of PPARα and β/δ called elafibranor. This study investigates its effects in mice fed a choline-deficient high-fat diet characterized by obesity and insulin resistance. In these mouse models, improvements in liver steatosis, inflammation, and fibrogenesis were demonstrated, and are associated with the following: a decrease in alpha smooth muscle actin (αSMA) and collagen type I alpha 1 (COL1A1) expression [120]; and elevated levels of epithelial–mesenchymal transition (EMT)-promoting protein calcium-binding protein A4 (S100A4). These changes are linked to PPARβ/δ activation and decreased cytokine signaling box containing protein 2 (ASB2) levels. This is a protein that regulates the degradation of S100A4 [121] (Table 1). Currently, there are more studies involving pharmacological treatments targeting PPARs, including the previously mentioned PPAR agonists, some of which are still ongoing or were halted [122,123].

**Table 1:** Summary of studies with PPARs as pharmacological targets.

Medication	Active Compound	Targeted PPAR	Population	Study Design/Method	Outcomes	Adverse/Side Effects
Lanifibranor [115] NATIVE trial	PPAR agonist	PPARα PPARδ Partial activation of PPARɣ	247 non-cirrhotic, highly active MASH * patients	Double-blind randomized controlled trial	Improvements in Triglycerides, HDL cholesterol, and insulin levels and steatosis	Gastrointestinal adverse events, peripheral edema, anemia
Lanifibranor [116]	PPAR agonist	PPARα PPARδ Partial activation of PPARɣ	247 MASH * patients with a poor cardiometabolic health	Clinical trial	Increased adiponectin levels Improvements in hepatic and cardiovascular health	Gastrointestinal adverse events, peripheral edema, anemia Weight gain of 2.5 kg
Icariin [117]	Flavonoid glycoside	PPARα	48 Murine models (rat) with type 1 diabetes	Experimental study	Normalization endothelial dysfunction. Inhibition of endoplasmic reticulum stress Activation of endothelial nitric oxide synthase	NA *
Daidzein [119]	Isoflavone	PPARα PPARγ	HUVECS *	In vitro experimental study	Reversed high glucose levels Amelioration of HUVECs * proliferative damage	NA *
Elafibranor [120]	PPAR agonist	PPARα PPARβ/δ	18 Murine MASH * models (mice)	In vivo and in vitro experimental study	Amelioration of steatosis and inflammation Increased (EMT) *-promoting proteins	NA *

* Metabolic-associated steatohepatitis (MASH), human umbilical vein endothelial cells (HUVECS), epithelial–mesenchymal transition (EMT).* Not applicable (NA): due to nature of study, no side or unwanted effects to report.

## 6. Discussion

Endothelial activation in MASLD involves the secretion of proinflammatory cytokines and adhesion molecules; this activation shifts the endothelial cells from a quiescent to an active state, promoting inflammation and endothelial dysfunction.

**Figure 2:**
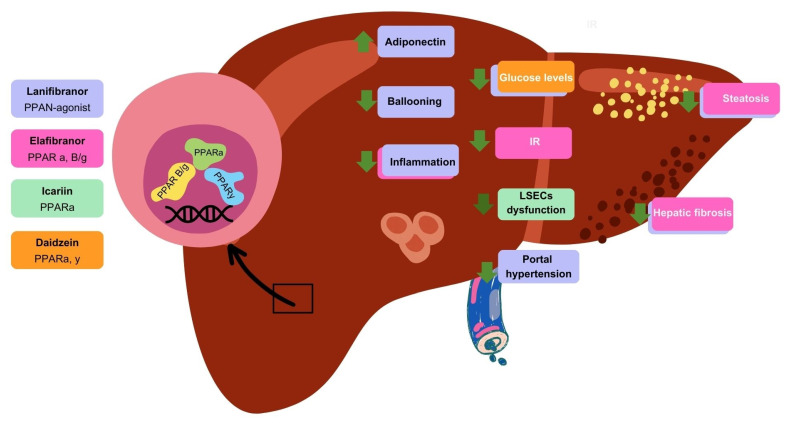
**Effect of PPAR agonists on MASLD pathology.** Through PPAR agonist drugs, there are different beneficial actions at the liver level for MASLD pathology. Lanifibranor, a PPAR agonist, reduces glucose levels and steatosis and improves portal hypertension and fibrosis; additionally, it increases adiponectin levels, which reduce hepatocyte ballooning and, consequently, inflammation. Elafibranor is a PPAR α-β/δ agonist that decreases inflammation and insulin resistance (IR), which synchronously slows down steatosis and fibrosis progression. Icariin, a PPARα agonist, reduces endothelial dysfunction thereby affecting liver sinusoidal endothelial cells (LSECs). Finally, Daidzein, a PPARα,γ agonist, promotes decreased levels of glucose.

PPARs are pivotal in modulating inflammation, particularly within the hepatic context (Figure 2). Specifically, PPARα exerts significant control over hepatic inflammation because it is predominantly abundant in the liver. The anti-inflammatory mechanisms include downregulating acute-phase genes, such as IL-Ra and NF-kB inhibitors, through transrepression, which in turn inhibits their transcriptional activity. This regulatory action is crucial for maintaining hepatic homeostasis. Notably, murine model research has demonstrated the DNA-binding domain mutations present in PPARα that limit its transcriptional activity and exhibit protection against liver inflammation and progression to fibrosis. This suggests that PPARα is capable of controlling both the initiation and resolution of hepatic inflammation. Furthermore, the activation of PPARα by synthetic fibrates was shown to have a beneficial impact on vascular function. For instance, fenofibrate, a PPARα agonist, enhances endothelial NO availability, which is crucial for vascular health. This effect underscores the potential therapeutic effects of PPARα activation in managing the endothelial dysfunction associated with MASLD.

Regarding PPARβ/δ, evidence suggests that it has an influence on lipid and fatty acid regulation and glucose metabolism. The study of PPARβ/δ agonists demonstrates their role in obesity and IR diseases, such as hepatic ischemia/reperfusion injury through NF-κβ pathway inhibition, lipid adipocyte accumulation, and diabetic osteoporosis.

Meanwhile, PPARγ plays a role in regulating endothelial activation through the modulation of NO activity, oxidative stress, and apoptosis by suppressing AP-1. PPARγ reduces the expression of VCAM-1 thereby mitigating endothelial activation. Additionally, PPARα and PPARγ activation suppresses endothelin-1, reducing chemotaxis and improving vascular function.

The significant role of PPARα, along with other PPAR isoforms, highlights the potential of PPARs as pharmacological targets in MASLD. All three PPAR isoforms may represent a promising approach for addressing the full spectrum of MASLD, from IR to liver fibrosis (Figure 2). This potential has been studied by Francque et al. with the pan-PPAR agonist lanifibranor, which led to improvements in both the metabolic and hepatic health aspects of MASLD patients. Additionally, its role in cardiovascular health amelioration was demonstrated [116].

Moreover, there are several ongoing clinical trials focusing on novel therapeutics. *Icariin* modulation in endoplasmic reticulum stress and the stimulation of NO synthase through the PPARα/Sirt1/AMPKα pathway can significantly improve vascular endothelial function in diabetes, which seems to be a key mechanism of IR [119]. On the other hand, daidzein, through its dual agonist activity on PPARα and PPARγ, could address T2DM-related dyslipidemia and vascular inflammation and mitigate glucose-induced damage in endothelial cells. Moreover, using elafibranor for treating MASLD by influencing EMT-related proteins, such as S100a4 and ASB2, could be possible via fibrogenic processes.

Notwithstanding, the studies cited have certain limitations. Firstly, some studies did not explore the potential adverse effects of PPARs agonists; secondly, small sample sizes and short administration/supplementation times might not be enough for observing significant changes in the pathology of MASLD. However, it is important to mention that at this point, clinical trials are either ongoing or halted. Therefore, a conclusive clinical hypothesis with a definitive and reliable background is not available. Furthermore, the dark side of molecular treatments cannot be ignored, due to all the known biological and cellular implications that this entails.

Finally, a combined analysis of animal models and clinical trials could provide valuable insights into the mechanisms of MASLD related to endothelial dysfunction and PPARs; however, researchers must carefully consider the limitations when interpreting the results through the extrapolation of animal data to human physiology because the heterogeneity of human populations compromises the development of an accurate predictive animal model for healthy, normal humans, as well as high-risk groups. Regrettably, inter-species extrapolation is still poorly understood and not applicable.

Moreover, the new definition of MASLD complicates its study because more underlying pathologies are included in diagnosis. This leads to a more complex scenario in which the discovery of an effective treatment should include an all-edges coverage of the entire disease spectrum. A more specific pharmacological treatment that perhaps includes multiple targets should be the focus, in addition to its combination with personalized medicine.

Future research directions that address unresolved questions in the field are mandatory. A deep study of the role of glycocalyx in MASLD is required, as well as basic and clinical studies that explore the molecular impact of ED in MASLD, which would lead to a more comprehensive evaluation of the management of and reduction in cardiovascular risks in liver disease patients. This integrated approach could help identify novel biomarkers and therapeutic strategies that enhance the prevention and treatment of comorbidities associated with metabolic syndrome, particularly in patients with MASLD.

## 7. Conclusions

The presence of ED in MASLD is an underlying pathological consequence that needs to be considered. PPARs are potential regulators of metabolism and inflammation, where the isoform PPARα is indispensable in the regulation of hepatic inflammation and is considered a key molecule for addressing metabolic dysfunction-associated steatotic liver disease pathogenesis. However, compensatory mechanisms among PPAR isotypes should be considered. PPARs are promising as potential therapeutic targets in the development of pharmacological agents for managing MASLD and associated vascular complications. Future research should continue to explore their potential and underlying mechanisms to optimize treatment strategies for MASLD and related metabolic disorders.

## Data Availability

Not applicable.
